# Quantitative characterization of viscoelastic behavior in tissue-mimicking phantoms and *ex vivo* animal tissues

**DOI:** 10.1371/journal.pone.0191919

**Published:** 2018-01-26

**Authors:** Ashkan Maccabi, Andrew Shin, Nikan K. Namiri, Neha Bajwa, Maie St. John, Zachary D. Taylor, Warren Grundfest, George N. Saddik

**Affiliations:** 1 Department of Electrical Engineering, University of California, Los Angeles, Los Angeles, CA, United States of America; 2 Center for Advanced Surgical and Interventional Technology, CASIT, Los Angeles, CA, United States of America; 3 Wellman Center for Photomedicine, Massachusetts General Hospital, Boston, MA, United States of America; 4 Department of Ophthalmology, Jules Stein Eye Institute, University of California, Los Angeles, CA, United States of America; 5 Department of Bioengineering, University of California, Los Angeles, Los Angeles, CA, United States of America; 6 Department of Head and Neck Surgery, David Geffen School of Medicine, Los Angeles, CA, United States of America; LAAS-CNRS, FRANCE

## Abstract

Viscoelasticity of soft tissue is often related to pathology, and therefore, has become an important diagnostic indicator in the clinical assessment of suspect tissue. Surgeons, particularly within head and neck subsites, typically use palpation techniques for intra-operative tumor detection. This detection method, however, is highly subjective and often fails to detect small or deep abnormalities. Vibroacoustography (VA) and similar methods have previously been used to distinguish tissue with high-contrast, but a firm understanding of the main contrast mechanism has yet to be verified. The contributions of tissue mechanical properties in VA images have been difficult to verify given the limited literature on viscoelastic properties of various normal and diseased tissue. This paper aims to investigate viscoelasticity theory and present a detailed description of viscoelastic experimental results obtained in tissue-mimicking phantoms (TMPs) and *ex vivo* tissues to verify the main contrast mechanism in VA and similar imaging modalities. A spherical-tip micro-indentation technique was employed with the Hertzian model to acquire absolute, quantitative, point measurements of the elastic modulus (E), long term shear modulus (η), and time constant (τ) in homogeneous TMPs and *ex vivo* tissue in rat liver and porcine liver and gallbladder. Viscoelastic differences observed between porcine liver and gallbladder tissue suggest that imaging modalities which utilize the mechanical properties of tissue as a primary contrast mechanism can potentially be used to quantitatively differentiate between proximate organs in a clinical setting. These results may facilitate more accurate tissue modeling and add information not currently available to the field of systems characterization and biomedical research.

## Introduction

Evaluation of viscoelastic properties of targets plays a crucial role in material science and medical diagnosis. Specifically, a target’s elastic modulus (*i*.*e*. stiffness) and long term shear modulus (*i*.*e*. viscosity, resistance to flow) can be determined from the resultant displacement from an applied force. The viscoelasticity of soft tissues is often associated with pathological state, and therefore can be utilized as a diagnostic tool for resections, amongst other operations, to reduce the morbidity associated with the disease at hand. Such a tool can particularly assist surgeons with tumor excision procedures, which could potentially improve clinical outcome [1–3]. Based on recent studies, tumorous tissues are typically characterized by an elastic modulus that differs from surrounding healthy tissue by several orders of magnitude [4, 5]. Such tissue contrast has been leveraged to delineate tissue margins between diseased and normal regions [4, 5]. Currently, standard clinical practice, especially in head and neck subsites, relies heavily on palpation in determining relative tissue stiffness [1, 2]. This method is based on a qualitative assessment of the region, and in many cases, the size and/or the location of the lesion makes this technique an insufficient method for medical diagnosis. A non-invasive, accurate, and high-resolution technique that uses viscoelastic properties of tissue to generate contrast may be more appropriate to detect small tissue abnormalities that are otherwise occult in palpation assessment.

Over the last decade, there has been growing interest in studying viscoelastic properties of soft tissues. The relation between tissue feature and tissue pathology has primarily been explored by vibroacoustography (VA) and acoustic radiation force in combination with various imaging modalities (*i*.*e*. Magnetic Resonance (MR) and Optical Coherence Tomography (OCT)). While both VA and similar techniques are relatively new research imaging approaches that are used to investigate and reconstruct mechanical properties of tissue, VA has gained more attention due to its relatively high resolution, minimal cost, and potential real-time imaging capabilities [6]. In comparison to palpation, the spatial resolution of VA is in the sub-millimeter range and the depth of penetration is in the sub-centimeter range, making the technique more suitable for high-resolution detection and imaging of tissue abnormalities [7].

In VA, the target absorbs an applied oscillating force and produces an acoustic emission field at the beat frequency, which is detected by a nearby, highly sensitive hydrophone [8]. Our group has previously reported on a novel VA imaging system that has been used to generate high contrast maps of tissues in response to a localized acoustic radiation force in both TMPs and *ex vivo* human squamous cell carcinoma tissues [3, 9, 10]. Although multiple studies have investigated VA to detect abnormal from healthy tissue regions with enhanced boundaries [7, 9, 11, 12], a direct correlation has yet to be established between acoustic signal and mechanical measurements [3]. Steps towards confirming viscoelasticity as the main contrast mechanism of VA require quantification of the major components of viscoelasticity, elastic modulus, long term shear modulus, and relaxation time constant (*i*.*e*. decay time), in clinically relevant models [3, 13]. To maintain clinical relevance, these models must incorporate static values of viscoelasticity to best mimic standard clinical practices, particularly palpation, which rely on static evaluation of tissue.

Moreover, existing imaging modalities and their respective theoretical models either fail to include significant mechanical tissue properties (*i*.*e*. elastic modulus, long term shear modulus, or relaxation time constant) or oversimplify the model with too many theoretical assumptions [14, 15]. Therefore, there is a need for an imaging modality with a complete model that more accurately characterizes the properties of tissue. Current models have been explored, and while each pose significant findings, none are yet suitable for tumor delineation in viscoelastic imaging modalities, such as VA. Phase-domain photoacoustic sensing is able to calculate absorption constants using temporally-delayed laser pulses by chirping modulation. This optical absorption constant is dependent on the mechanical properties of the sample, more specifically density and heat capacity, as the sample thermally expands upon exposure to laser [16]. Modeling using photoacoustics has also led way to quantitative photoacoustic tomography (qPAT), which generates a map of tissue response to light energy absorption. Although conventional models of qPAT, particularly diffusion approximation and radiation transport, have shortcomings, a novel model utilizing Monte Carlo simulations proves promising, as generated images possess high contrast [17]. Both photoacoustic models place emphasis on optical absorbance of tissue, which suits the respective technologies very well, and serve as paradigms for future modeling. However, these models do not incorporate viscoelastic properties (*i*.*e*. elasticity, long term shear modulus, and time constant) to the extent that must be quantitatively determined for applications in intra-operative surgical procedures and development of viscoelastic imaging modalities such as VA.

Personalized cardiac biomechanical modeling, in relating kinematics to dynamics through patient-specific modeling, is an additional area that has been explored for mechanical modeling of tissue pathology. Simulated mechanical characterization of the left ventricle has presented novel surrogates for rheological and non-invasive imaging measurements. Accurate modeling of the left ventricle’s cardiac cycle using a Holzapfel-Ogden model was successfully implemented with paralleled MR images as validation [18]. The simulated measurements were calculated from sample-specific data including fiber orientations, ventricle cavity volume, and overall geometry of the ventricle. However, this model explicitly assumed that the myocardial tissue behaved as incompressible hyper-elastic material, and mandated robust model selection and parameterization that did not include viscoelastic-specific parameters.

Utilizing energy-based cost function to identify biomechanical frameworks of biological tissue is yet another promising approach in material characterization. However, this method has not yet demonstrated the ability to quantitatively characterize parameters of interest, particularly long term shear modulus and time constant, in more relevant clinical settings [19].

On the contrary, shear wave elastography developments by Kazemirad *et al*. may provide promising techniques in quantitative viscoelastic characterization. Loss modulus (*i*.*e*. viscosity) and storage modulus (*i*.*e*. elasticity), are calculated through this method, which indirectly provides estimation of mechanical properties of soft biological tissues for characterizations in medical applications [20]. Unfortunately, the current proposed model is frequency-dependent and is in terms of propagation distance when analyzing the cylindrical shear wave field produced by the homogeneous tissue. This further demonstrates that a more thorough methodology is still needed to provide valid and accurate mechanical characterization of tissue.

The work presented in this paper originates from our previous studies that correlated elastic properties of TMPs with radiation force intensity measurements from a VA system to develop a model to explore the observed behavior of targets under the acoustic radiation force [7]. Based on this previous correlational study between VA signal intensity and elastic moduli in TMPs, we concluded that additional parameters, specifically long term shear modulus and relaxation time constant, should be considered for more accurate modeling of viscoelastic properties of tissues. Herein, we use static rheological models, specifically the Hertzian model (see Appendix), to characterize mechanical properties of pre-clinical targets. This model was used in conjunction with micro-indentation technique to acquire absolute measurements of mechanical properties of TMPs and *ex vivo* animal tissues. Mechanical measurements were acquired in three types of TMPs, specifically agar, gelatin, and polyvinyl alcohol (PVA), at concentrations that mimic the acoustic properties of human tissue. A total of 10 measurements, 5 for each depth, were conducted on each phantom type.

Liver and gallbladder tissue from a porcine model and liver tissue from a rat model were also chosen to more closely mimic human tissues. A total of 13 liver samples and two gallbladder samples from two different porcine subjects were examined. *Ex vivo* indentation measurements consisted of a total of 34 porcine liver indentation measurements, 17 for each depth, and 7 measurements of porcine gallbladder tissue at one depth due to the tissue’s small thickness. This viscoelastic characterization test was also performed on 14 samples of rat liver tissue from three different subjects, resulting in 38 total measurements, 19 for each depth. A static, spherical-tip micro-indentation technique produced elastic modulus, long term shear modulus, and time constant values for *ex vivo* animal tissue and TMPs. The results were then compared and assessed for statistical significance. These results could be used in future studies that investigate the contrast mechanism of dynamic imaging techniques like VA and similar acoustic radiation techniques.

## Modeling and methods

**Fig 1:**
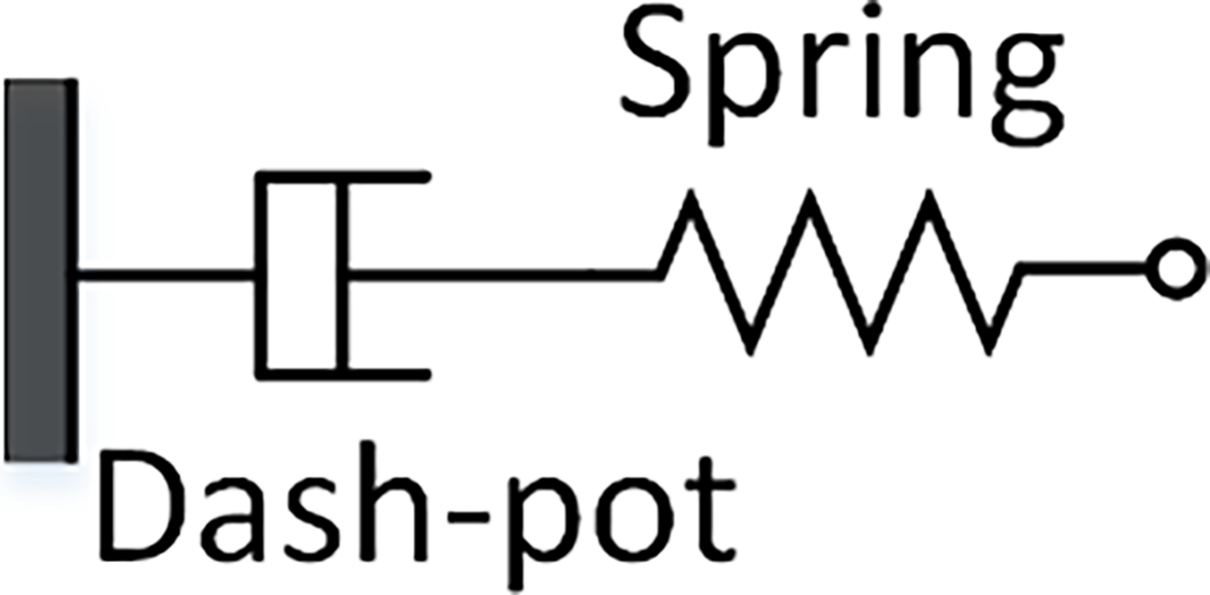
Viscous Dash-pot and linear spring lumped element model connected in series.

Biological tissues are modeled as viscoelastic materials due to the manifestation of hysteresis in their stress relaxation behavior [21, 22]. The word viscoelastic is a combination of viscous fluidity and elastic solidity, and thus, biological materials under stress and strain exhibit both viscous and elastic behavior. In modeling of materials, such as biological tissues, the linear elastic Hookean spring, which describes elasticity behavior, and the linear viscous Newtonian Dash-pot, which describes long term shear modulus behavior, are used in conjunction to examine and understand the performance of these materials under spring force and displacement. Fig 1 shows the linear elastic spring and linear viscous Dash-pot [21, 22].

The linear elastic spring relates stress, defined as the exerted force per unit area, to strain, defined as changes in length with respect to initial length, in a linear fashion by the elastic modulus (*E*). During this mechanical process, the material undergoes an instantaneous deformation upon loading and an instantaneous de-straining upon un-loading. Eq 1 illustrates the simplified relationship in a one-dimensional (1D) case:

σ=Eε (1)

where σ and ε represent stress and strain in 1D, respectively.

The linear viscous Dash-pot contains a piston-cylinder filled with a viscous fluid and, by definition, it linearly relates stress and strain by the long term shear modulus of the material (η). Eq 2 represents the linear elastic spring and linear viscous Dash-pot:

$$\sigma =\eta \;\dot{\varepsilon }$$ (2)

Multiple models, such as Maxwell Dash-pot, Kelvin-Voigt, and Hertzian, have been used to characterize the mechanical properties of biological tissues [21]. Of these three models considered for this paper, the Hertzian model was chosen to analyze the acquired data because a spherical-tip micro-indenter was used. As shown by Oyen, Hertzian model offers a versatile, depth-sensing indentation technique with spherical-tip indenters to predict accurate viscoelastic mechanical behavior of biological tissues [23]. Moreover, in this model, linear elastic deformation of both shapes is assumed with a quadratic pressure distribution along the area of contact and assumes only elastic deformation [24]. It also has previously been used for other biological spherical-tip micro-indentation experiments, including characterizing bovine ocular tissues [25]. The derivation of the model is included in the appendix.

### Target preparations and experimental setup

Three TMP types, agar, polyvinyl alcohol (PVA), and gelatin, were fabricated and investigated. These materials were chosen to mimic relevant human anatomical structures (*i*.*e*. prostate, oral cavity, liver, and breast) in terms of acoustic and mechanical properties [10–12, 18–21]. The homogeneous TMPs were synthesized in molds with defined geometries. Due to the controlled phantom synthesis, the mean phantom thickness, measuring ~18 mm, did not vary among each phantom.

**Fig 2:**
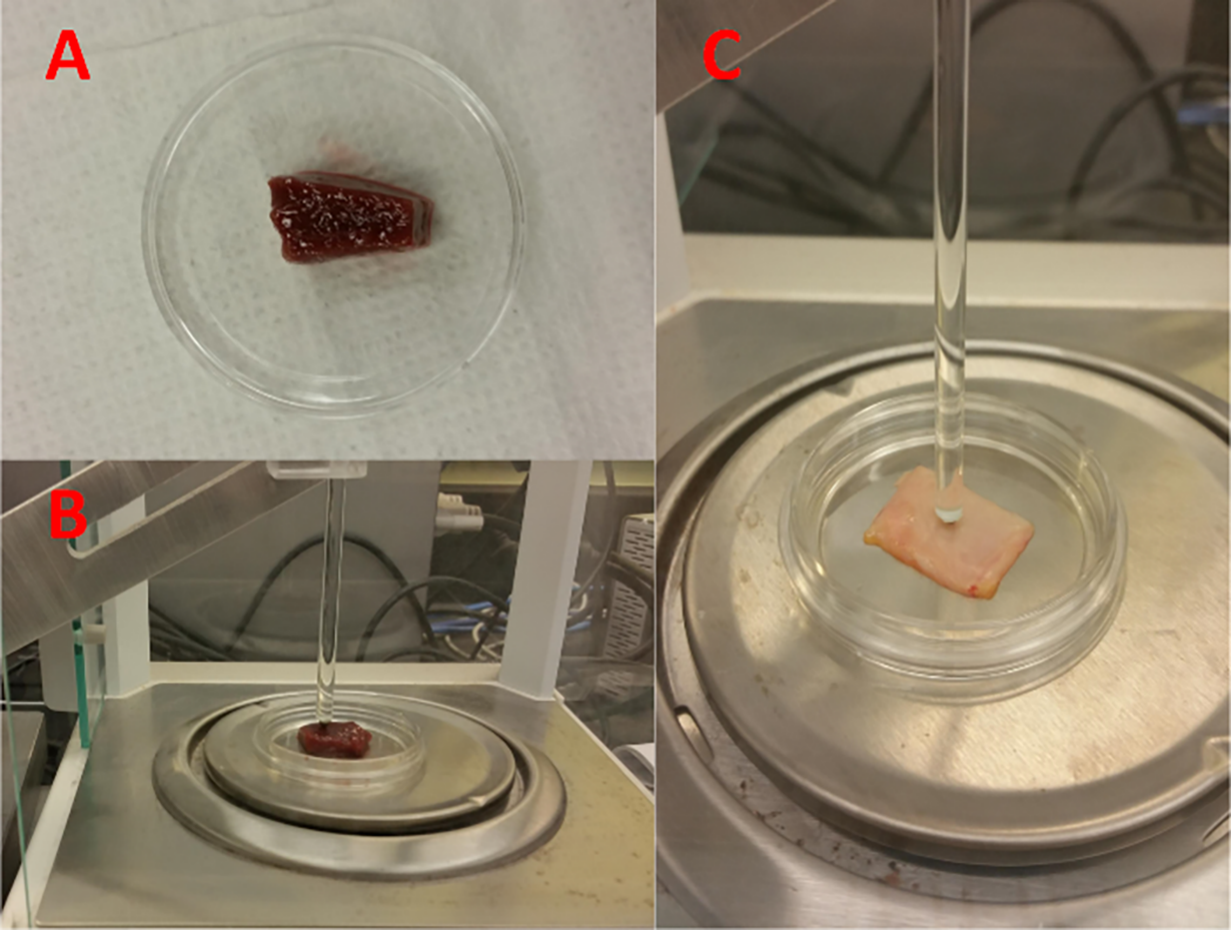
Fig 2A displays an excised porcine liver sample placed in a petri dish prior to indentation, while Fig 2B illustrates the same porcine liver specimen undergoing the micro-indentation test. Fig 2C illustrates the porcine gallbladder specimen undergoing the micro-indentation. Spherical tip diameter was adjusted due to the thickness of the specimen.

Fresh *ex vivo* liver and bile ducts were harvested from porcine and male Dewey rats shortly after each animal was euthanized under protocols #2009-094-23 for rat tissues and #2003–093 for porcine tissues. Since the *ex vivo* tissues were freshly excised, their thicknesses and geometrical arrangements were not identical to one another. The thicknesses were measured as the following: ~11 mm for porcine liver, ~6 mm for rat liver, and ~1 mm for porcine gallbladder. These excised samples were relatively flat and of similar lateral dimensions to avoid collection/slippage error. The fabrication process, rationale, and procedure for the TMPs and *ex vivo* tissues are attached in the appendix. Fig 2 illustrates the experimental set-up for *ex vivo* animal specimen undergoing micro-indentation.

A 100 nm precision linear stepper motor and controller (LNR50 Series, Thorlabs, Newton, NJ) were synchronized with a 100 μg precision analytical balance (ML Model, Mettler-Toledo, Columbus, OH) to perform the viscoelastic measurements on the targets [25]. The stepper motor was connected to an acrylic rod that displaces a stainless steel sphere. A 2 mm diameter sphere was used to create an indentation depth of 300 μm and 400 μm for *ex vivo* porcine liver samples, 200 μm and 300 μm for *ex vivo* rat liver, and 100 μm for porcine gallbladder. Porcine gallbladder was only indented at one depth due to its very small thickness. A slightly larger, 4 mm diameter sphere was used to create indentation depths of 600 μm and 800 μm for all TMP targets. Two indentation depths were utilized to accurately compute viscoelastic behavior of each target and to further illustrate the stability and consistency of the measurement parameters.

For all micro-indentation measurements, the indentation depth was much less than the radius of the sphere to be in the theoretical regime of contact mechanics between sphere and flat surface. These indentation distances were also chosen based on the thickness of the targets to stay within the linear regime, ~3% strain rate, of the samples [24, 26]. The Hertzian model assumes indentation of up to 3% of the specimen’s thickness to be negligible in comparison to sample thickness, so that the substrate does not influence the calculations [27]. Every indentation was performed in a different location; therefore, pre-stress and timing constraint between consecutive indentations were not considered. The displacement depth for all targets, except gallbladder tissues, was less than the radius of the indenter to avoid any subsequent errors in the recordings.

**Fig 3:**
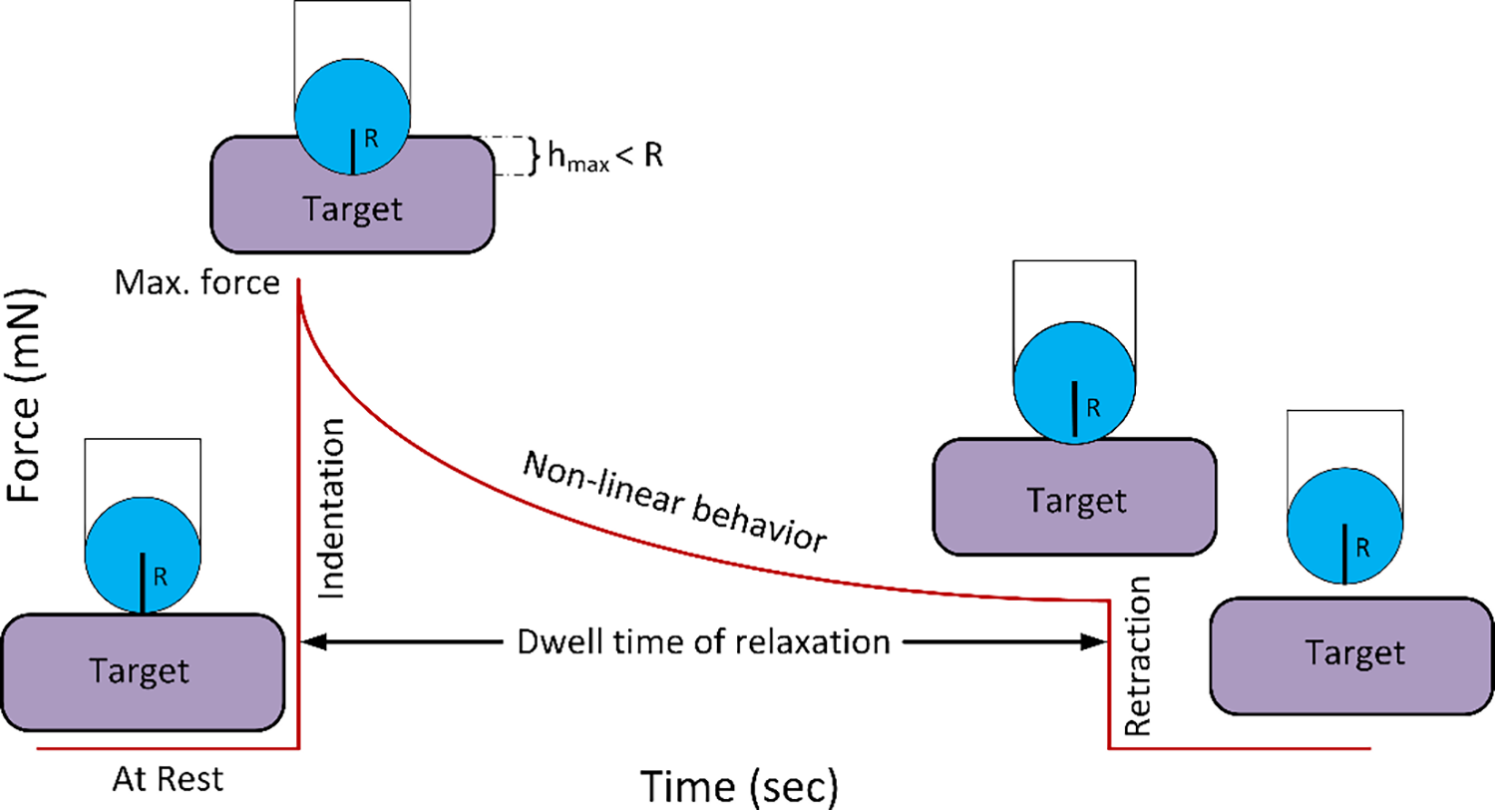
Spherical-tip micro-indentation experimental set-up. As illustrated in the figure, the sphere induces indentation, smaller than its radius, into the target (Indentation period). After it reaches the maximum depth, it allows the target to relax, with regards to exerted force, in a period of 300 seconds (dwell time of relaxation period). After completion of the process, the sphere is moved away from the target (Retraction period).

The indenter displaced downward against the targets, which were placed on an analytical balance pan. This balance served to record the force measurements. Prior to each measurement, the balance was zeroed to avoid surface tension errors, which are transiently negative values upon initial contact of the probe with the liquid layer of the tissue [25]. Shortly after, the target was indented by the sphere, at 2 mm/sec motor speed, generating a positive force by the target, conveying that contact has begun. Once the given displacement was reached, the indenter remained fixed in position, and the target was allowed to relax for approximately 300 seconds. During this period, the balance beneath the target recorded the applied force from the target to the sphere. Specimens were periodically moisturized using Ringer’s lactate solution throughout the entire experimental procedure. Moreover, in order to minimize changes in load due to evaporation of water surrounding the specimen, the load cell was surrounded by a glass closed-chamber on all sides except for a small slit for the indenter shaft. Fig 3 illustrates the experimental setup that was used to acquire measurements.

## Results

**Fig 4:**
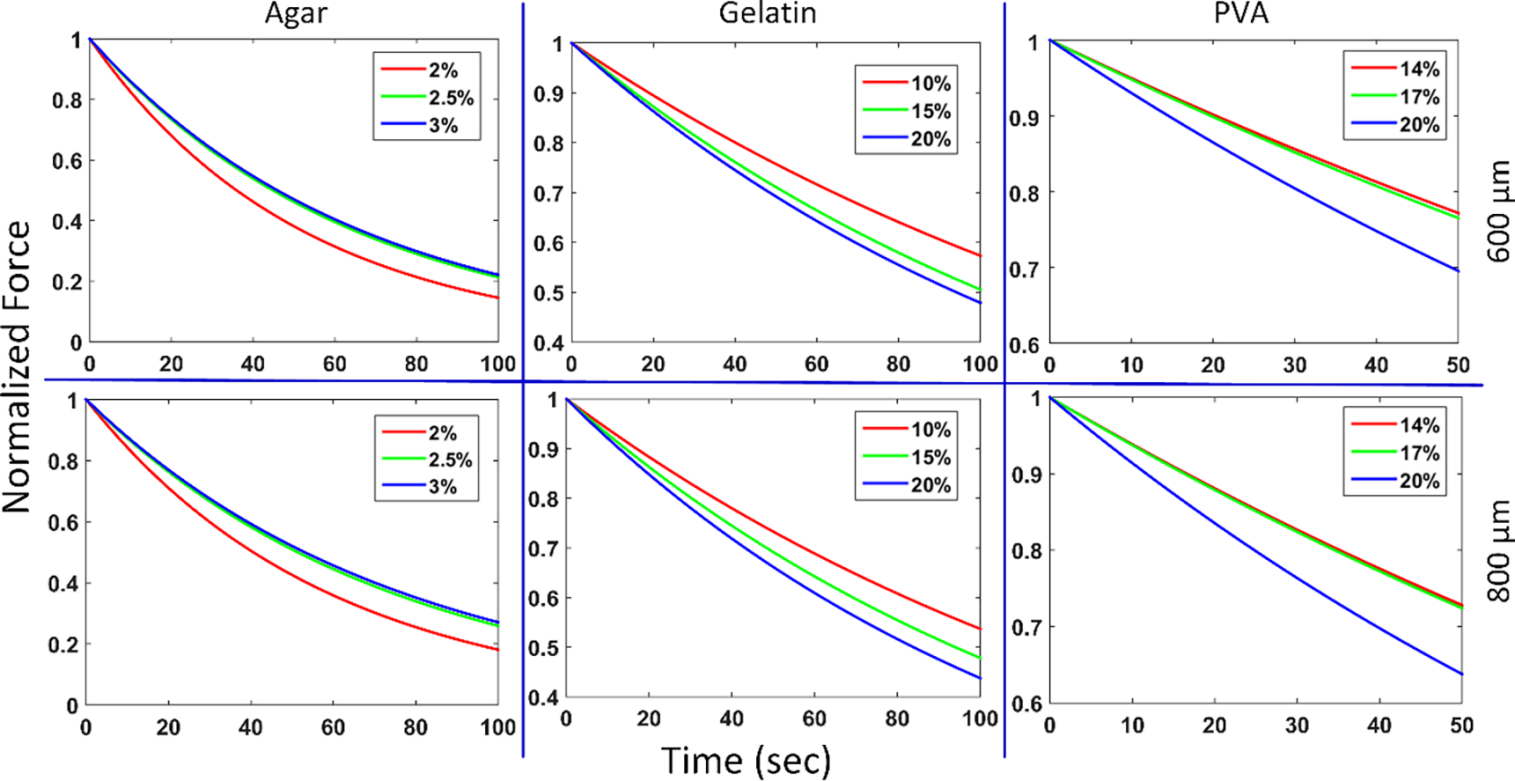
Mean elastic relaxation behavior plots of agar, gelatin, and PVA TMPs as a function of time. The top and bottom rows illustrate the 600 μm and 800 μm indentation behaviors, respectively. As shown, gelatin and PVA have the same relaxation behavior, but the opposite is observed for agar TMPs.

The viscoelastic behavior of TMPs and *ex vivo* porcine and rat tissues were examined by a micro-indentation technique using a stainless steel sphere. Fig 4 illustrates the relaxation plots for all TMPs and Table 1 shows the instantaneous elastic modulus, analogous to Young’s modulus, long term shear modulus, and time constants with standard deviation of the mean for all TMPs at the two different indentation depths. Elastic modulus and long term shear modulus values were calculated by fitting the collected data to a first order exponential decay [3]. From this function, the time constants were calculated using the single relaxation curve in MATLAB. The relatively small standard deviation of the mean for elastic modulus and long term shear modulus measurements of each phantom type shows a very small deviation from the mean in both indentation depths for all phantoms.

**Table 1:** Mean elastic modulus, long term shear modulus, and time constant data, including standard deviation of the mean, for each type of TMP: PVA, gelatin, and agar.

Phantom Type	Elastic Modulus (kPa)		Mean (kPa)	Standard Deviation	Long term Shear Modulus (MPa sec)		Mean (MPa sec)	Standard Deviation	Time Constant (sec)
	600 μm	800 μm			600 μm	800 μm			
**14% PVA**	5.722	5.598	5.660	0.158	1.105	0.883	0.994	0.040	175.657
**17% PVA**	9.552	9.319	9.435	0.200	1.785	1.467	1.626	0.075	172.330
**20% PVA**	33.563	33.866	33.715	1.015	4.693	3.824	4.259	0.273	126.317
**10% Gelatin**	15.823	16.873	16.348	0.897	2.830	2.710	2.770	0.128	169.424
**15% Gelatin**	43.518	46.903	45.210	2.243	6.390	6.358	6.374	0.309	140.988
**20% Gelatin**	62.010	69.907	65.959	3.087	8.649	8.957	8.803	0.445	133.458
**2% Agar**	106.497	102.778	104.638	3.911	5.595	6.000	5.797	0.210	55.405
**2.5% Agar**	137.034	133.918	135.476	4.647	9.122	10.045	9.583	0.607	70.737
**3% Agar**	199.049	191.284	195.166	4.281	13.575	14.913	14.244	0.840	72.984

As shown by the calculations in Table 1 and Fig 4, as the concentration of gelatin and PVA TMPs increases, the calculated time constant decreases, but in the case of agar TMPs, the exact opposite was observed. This result may be due to the structural properties as well as the structural cross-linking of the agar substituents within the phantom.

**Fig 5:**
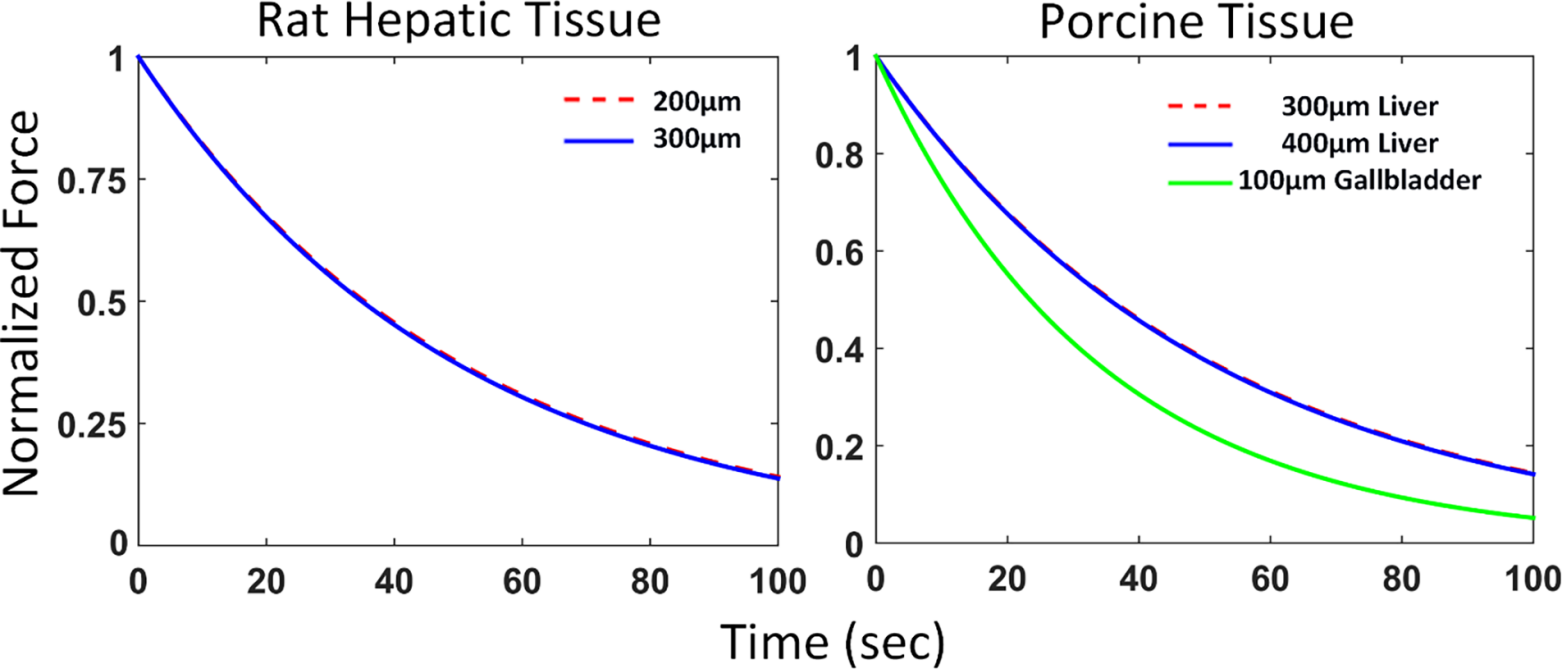
Mean elastic relaxation behavior plots of *ex vivo* animal tissues. The liver tissues for both rat and porcine models decay exponentially in a similar fashion for both indentation depths. The relaxation behaviors of the porcine gallbladder and liver tissue are very distinct, illustrating their unique viscoelastic characteristics.

Fig 5 illustrates the relaxation plots and Table 2 shows the instantaneous elastic modulus, long term shear modulus, and time constant for *ex vivo* porcine liver, porcine gallbladder, and rat liver. All *ex vivo* tissues had small variations, as shown by their respective standard deviation of the mean in Table 2.

**Table 2:** *Ex vivo* porcine liver, porcine gallbladder, and rat liver elastic modulus, long term shear modulus, and time constant are calculated. Standard deviation of the mean for each tissue is also computed.

Tissue Type	Depth	Elastic Modulus (kPa)	Mean (kPa)	Standard Deviation	Long term Shear Modulus (MPa sec)	Mean (MPa sec)	Standard Deviation	Time Constant (sec)
**Porcine Liver**	300 μm	2.575	2.553	0.085	0.138	0.135	0.006	52.879
	400 μm	2.531			0.132			
**Rat Liver**	200 μm	2.890	2.758	0.094	0.154	0.147	0.007	53.299
	300 μm	2.626			0.140			
**Porcine Gallbladder**	100 μm	4.730	4.730	0.735	0.176	0.176	0.033	37.209

During gallbladder tissue preparation, excess bile was removed from the tissue to avoid slippage of the sphere indenter, which could have potentially affected the viscoelastic measurements. The standard deviation of the mean for both rat and porcine liver were very small due to consistency of the data; however, that error value was a bit higher for the gallbladder. This could be due to the remnants of bile that could not be removed, and the small tissue thickness of the gallbladder samples. In comparison to the liver tissues, the porcine gallbladder tissues demonstrate a smaller time constant, though a higher elastic modulus and long term shear modulus. Two-tailed unequal variance (heteroscedastic) T-tests were conducted for TMPs and *ex vivo* tissues, the results of which are illustrated in Table 3.

**Table 3:** Two-tailed T-test statistical analysis of elastic modulus and long term shear modulus of TMPs and *ex vivo* tissue specimen (α = 0.05). The Small Depth corresponds to the lower indentation depth measurement of each tissue, while the Long Depth corresponds to the higher. Porcine gallbladder was only indented at one depth due to its small thickness.

Subject Type	Depth	P-Value (α = 0.05)	
		Elastic Modulus	Long Term Shear Modulus
**14% vs. 17% PVA**	600 μm	0.00002	0.00005
	800 μm	0.00001	0.00343
**17% vs. 20% PVA**	600 μm	0.00013	0.00091
	800 μm	0.00002	0.00040
**14% vs. 20% PVA**	600 μm	0.00013	0.00188
	800 μm	0.00002	0.00044
**2% vs. 2.5% Agar**	600 μm	0.01038	0.01720
	800 μm	0.00553	0.00145
**2.5% vs. 3% Agar**	600 μm	0.00017	0.03186
	800 μm	0.00001	0.00582
**2% vs. 3% Agar**	600 μm	0.00001	0.00318
	800 μm	0.00001	0.00082
**10% vs. 15% Gelatin**	600 μm	0.00017	0.00043
	800 μm	0.00008	0.00011
**15% vs. 20% Gelatin**	600 μm	0.00826	0.02962
	800 μm	0.00037	0.00560
**10% vs. 20% Gelatin**	600 μm	0.00011	0.00035
	800 μm	0.00001	0.00005
**Rat Liver vs. Porcine Liver**	Small	**0.10364**	**0.27704**
	Long	**0.57440**	**0.53586**
**Rat Liver vs. Poricne Gallbladder**	Small	0.04657	**0.54677**
**Porcine Liver vs. Porcine Gallbladder**	Small	0.02604	**0.29875**

The elastic modulus and long term shear modulus were significantly different among varying concentrations for each type of TMP. Hence, our mechanical testing methodology is evidently capable of producing distinct results for different types of viscoelastic material. Furthermore, there was no statistical significant difference between rat and porcine liver in both elastic and long term shear modulus, illustrating that liver possesses similar viscoelastic properties despite being from different species. Within the same animal, different organs were distinguished in terms of viscoelastic properties, as seen by the statistical significant difference between porcine liver and gallbladder elastic modulus. However, the long term shear modulus between the liver and gallbladder tissues were not significantly different.

## Discussion

This paper characterizes the viscoelastic properties of TMPs and *ex vivo* animal tissues by examining elastic modulus, long term shear modulus, and relaxation time constant in a unique fashion. The Hertzian mathematical model and spherical-tip micro-indentation were used to analyze the observed relaxation curves. The generated relaxation curves were fitted to a first order exponential fit with a relatively high R^2^ value (*i*.*e*. > 0.9) for all cases. Based on our results and statistical analysis, elasticity, long term shear modulus, and relaxation time constant should be considered for mechanical modeling of biological tissue with viscoelastic behaviors.

The TMP elasticity measurements show three unique elastic moduli ranges: PVA: 1–40 kPa (low), gelatin: 10–100 kPa (medium), and agar: 100–200 kPa (high). Along with their corresponding long term shear modulus values, the TMPs were examined to cover a wide range of viscoelasticity for soft tissues, also previously shown by Wells and Liang [4]. The similarity in acoustic property trends between the selected TMPs and human tissues (see Appendix) makes this study a crucial stepping stone for imaging modalities like VA. Moreover, due to our unique experimental set-up and measurement calculations, direct comparisons are not forthright for each aspect of the TMP study. However, similar approaches have been made. Hamhaber *et al*. completed a quantitative comparison of a shear wave MR elastography technique with mechanical compression tests on agar-agar gel phantoms. This was done by comparing strain-generated wave amplitudes from shear-wave MR elastography to computed shear modulus, and performing dynamic mechanical compression tests in the range of 125–400 Hz [28]. They computed the elastic modulus of their 2% agar-agar elastic modulus by making an assumption of Poisson ratio of incompressible materials. Their results were within the same range of ours. Furthermore, Pavan *et al*. examined non-linear elastic behavior of gelatin and agar phantoms by using Bose Endura TEC 3200 ELF system [29]. They used large oscillatory deformations (~25% strain rate) on 2-month-old phantoms samples, whereas the phantoms used in our study were freshly made and experiments were conducted on the same day with a strain rate of ~3%. They reported higher values than those in this study, which could be due to the age of their phantoms along with their high deformation rates. However, the overall trend demonstrated in our study showed higher concentrations lead to higher elastic modulus values, similar to the trend generated from the study by Pavan *et al*. Additionally, elasticity measurements on TMPs performed by Chen *et al*. were conducted using uniaxial compression, though the accompanied rheological model was not specified, which may explain discrepancies in results. The group reported significantly higher elastic moduli for 2% agar, with values varying between 250–650 kPa depending on strain rate [30]. This may be because the group applied extreme strains of up to 1.0 on their samples, and utilized measurements from regions of up to 0.4 strain, whereas the strain utilized in our study was 0.03 to remain within the linear regime of the specimens.

Long term shear modulus and time constant characterization were other features that were analyzed. To the best of our knowledge, there are no straightforward comparisons with the literature, but there are related works on long term shear modulus of soft tissues and solids characterizations. Catheline *et al*. performed measurements of viscoelastic properties of bovine muscle using transient elastography by comparing Maxwell Dash-pot to Voigt model [31]. Kobayashi *et al*. also performed a similar study on viscoelastic solids with high long term shear modulus by uniform shear stress deformation method in a temperature controlled setting [32]. However, the long term shear modulus values reported by Catheline *et al*. are orders of magnitude smaller, while Kobayashi *et al*. presents values that are orders of magnitude higher, than the ones reported on TMPs in this paper. The different deformation methods along with different implemented assumptions could be reasons for these discrepancies. Kobayashi *et al*. used pure shear and mainly tensile (extensional) deformation mode, while Catheline *et al*. used transient elastography with frequency ranges 50–350 Hz in 25 Hz step sizes. Although both methods may be valid in their own respects, they do not seem ideal for applications in characterizing tissue pathology. Unlike the aforementioned studies, our p-values were below 0.05 among all TMP paired T-tests, for both elasticity as well as long term shear modulus. Our study is one of few to demonstrate reliability and precision in all viscoelastic characterizations. Moreover, spherical-tip micro-indentation may be the step forward for correlating tissue viscoelasticity and acoustic response.

Porcine liver and gallbladder tissues, along with rat livers, were tested using the same technique that was used for TMPs. The tissues were excised fresh, immediately after the animals were euthanized, and kept in a saline solution during transport to maintain tissue integrity. Many scientists use pre-conditioning techniques with cyclic deformations for their *ex vivo* sample preparations. However, this technique was not used in this study because the generated results were already in steady-state mode and the elastic modulus, long term shear modulus, and time constant calculations were consistent with very low standard deviation of the mean [33]. Moreover, the duration of examination for each sample was relatively short, ~15 minutes per sample, demonstrating the perseverance of the physiological conditions throughout the span of the measurement.

Similar to our TMP viscoelasticity results, there is no direct comparison for our generated *ex vivo* porcine and rat tissue viscoelasticity data within the literature, but some methodologies have been developed. Kerdok *et al*. characterized the effects of perfusion on the viscoelastic characteristics of porcine liver by using indentation devices to measure the organ’s creep response to applied loads [34]. Their calculated time constants for both porcine and rat liver were very similar to the ones calculated in this study, demonstrating similarity in physiological structure as well the accuracy of our measurements. Elastic modulus and long term shear modulus were not directly calculated in their study, but the calculated time constant can alone be a precursor to the viscoelastic measurements since in theory, the time constant is the product of elastic modulus and long term shear modulus, as seen in Eq 13 of the Appendix.

Venkatesh *et al*. and Chen *et al*. reported healthy *in vivo* human parenchyma, rat parenchyma, and porcine liver shear modulus and long term shear modulus values using Magnetic resonance elastography for human and rat parenchyma liver and Shear wave Dispersion Ultrasound Vibrometry for the porcine liver [35, 36]. For comparison with these studies, since liver is assumed as an incompressible material, the Poisson ratio was estimated to be 0.50 [4], and the calculated elastic moduli using the relationship in Eq 3 was ~6.16 kPa for human, ~6.58 kPa for porcine, and ~5.26 kPa for rat.

$$G=\frac{E}{2*(1+\nu )}$$ (3)

However, both our calculated elastic modulus and long term shear modulus values for porcine and rat livers are smaller than the values reported by Venkatesh *et al*. and Chen *et al*. One reason for this discrepancy could be due to the assumptions (*i*.*e*. tissue homogeneity, density, the implemented model (Voigt model)), as well as the detection scheme they used for calculating these values. Since micro-indentation was used in this study, some of these assumptions, particularly density and homogeneity, can be ignored, even though liver tissues are not homogeneous [37]. Moreover, placing a tissue on a hard surface induces external forces (*i*.*e*. stress) that are not present in the *in vivo* state. Other factors, such as capsules, blood flow, cellular association, amongst other *in vivo* conditions, need to be taken into account for accurate measurements. However, our measurements, for both rat and porcine liver, were free of these potentially confounding *in vivo* factors. As depicted in Table 3, there was no statistical significant difference between rat and porcine liver in both elastic and long term shear modulus. This further illustrates that liver possesses similar viscoelastic properties despite being from different animals. Also, the time constants and relaxation curves for both rat liver and porcine liver were very similar, and statistically insignificant (*i*.*e*. p-value >0.05), conveying that the viscoelasticity of liver is generally similar between these two animals.

Porcine gallbladder was the third tissue type characterized in this study. We sought to use the viscoelastic properties of the gallbladder to differentiate it among other close organs, particularly the porcine liver. As presented in Table 2, the elastic modulus was ~2 kPa higher than the liver and there was a difference of ~20 seconds in the time constants. The p-value is also significant when comparing elastic modulus of porcine gallbladder to that of both rat liver and porcine liver. Thus, there exists a distinct difference in viscoelastic properties between the two porcine tissue types, liver and gallbladder. Even though the two organs are relatively close to one-another, their respective viscoelastic properties can be used to differentiate the two, highlighting clear boundary distinction between the two tissue types. However, the long term shear modulus between the liver and gallbladder tissues were not significantly different, likely due to the relatively limited sample size of gallbladder specimen, small thickness of gallbladder, and remnants of excess bile on gallbladder tissues. Aside from the future work needed to fully characterize gallbladder long term shear modulus, our mechanical testing methodology is capable of producing distinct viscoelastic characterizations with statistical significance for different tissue types. Future studies will include comparison between static (*i*.*e*. micro-indentation) and dynamic measurements of viscoelasticity properties of tissues.

These results can be used to facilitate validation studies of viscoelastic imaging techniques with the aim of investigating mechanical properties of tissue as the primary source of image contrast to develop a mathematical model. Furthermore, complete dynamic mechanical characterization, particularly those which assess stress and strain rates that bring the tissue to failure, in both *in vivo* and *ex vivo* cases, are critical for establishing mathematical models. These properties can then be used in conjunction with imaging modalities (*i*.*e*. VA) to accurately and precisely describe viscoelastic mechanical behavior of targets for accurate identification, border detection, and system characterization in the field of medicine.

## Conclusion

Accurate characterization and modeling of tissue still requires clinically relevant TMPs and fresh biological tissues. Given that biological tissues behave as viscoelastic materials, long term shear modulus, relaxation time constant, and elasticity must be considered in their evaluation. This study focuses on characterization of the viscoelastic properties of TMPs and *ex vivo* animal tissues; however, direct characterization and evaluation with *ex vivo* and possible *in vivo* biological organs in a dynamic fashion is still a necessity for further validation of mechanical properties. The experimental results in this study may bolster the possibility of using tissue mechanical properties, particularly viscoelasticity, as the primary contrast mechanism for developing new imaging modalities, like vibroacoustography (VA) and similar techniques. To this end, insights gained from assessment of animal tissues have helped researchers better understand the underlying mechanical behavior of biological tissue, but better *ex vivo* experimental setting (*i*.*e*. closer replication to non-homogeneous *in vivo* physiological parameters) must be implemented for optimal viscoelastic characterizations. Nevertheless, characterization and evaluation of *ex vivo* animal hepatic and gallbladder tissues under micro-indentation techniques, among other mechanical techniques, may furnish additional information that can guide researchers and scientists in modeling and investigative approaches for tissue response under a static force in target imaging (*i*.*e*. VA and similar techniques) and material characterization.

## Appendix

### Hertzian model

The solution for the viscoelastic spherical indentation relates the exerted force, *F*, from a rigid sphere with a radius, *R*, to the elastic modulus, *E*, and the Poisson ratio, ν, of an incompressible material at a given displacement, *h*, shown by Eq 4, where the viscoelastic counterpart of the Hertzian problem in elasticity is deduced from the elastic solution proposed by Hertz [38]. In this solution, the viscoelastic operators are substituted for elastic constants in the elastic solution, which allows for an identification of relaxation time constant behavior of specimens in terms of elastic modulus, Poisson ratio, indentation depth, and short and long-term shear elastic moduli. This solution is based on a single relaxation curve fit and utilizes a “ramp correction factor” (RCF) approach to correct the difference between ramp and step loading cycles for each exponential decay.

$$F=\frac{4E\sqrt{R}h^{3/2}}{3(1-\nu^{2})}$$ (4)

The Poisson ratio, the ratio of the transverse contracting strain to the elongation strain, of incompressible biological materials is assumed to be between 0.45 to 0.50. However, Poisson ratio was chosen to be 0.50, a common approximation used in literature for all TMPs and *ex vivo* animal tissues in this study [39, 40].

The relaxation response of a step-load (*i*.*e*. ideal), rigid, spherical-tip indenter to the material as a function of time and shear modulus, G (t), is illustrated by Eq 5:

$$F_{ideal}(t)=\frac{8\sqrt{R}}{3}h_{0}^{3/2}G(t)$$ (5)

where time-dependent shear relaxation modulus, G(t), is equal to E(t)/3. The observed rise time (*t*_*R*_) in real instances is not an instantaneous step loading process, as opposed to the ideal cases; hence the ramp correction factor (RCF) is used. Therefore, the viscoelastic integral operator for relaxation, where *u* is a strain function in terms of τ is used and is shown by Eq 6:

$$F_{real}(t)=\int_{0}^{t}G(t-\tau )[\frac{du(\tau )}{d\tau }]d\tau$$ (6)

By combing Eqs 5 and 6, the real exerted force as a function of time, F_real_ (t) is:

$$F_{real}(t)=\frac{8\sqrt{R}}{3}\int_{0}^{t}G(t-\tau )[\frac{d}{du}h^{3/2}(u)]du$$ (7)

As shown by Mattice et al.[41], the Boltzmann integral method is used to solve Eq 7 and for ramp-loading rate, k, displacement for ramp-hold relaxation can be written as:

$$h(t)=kt\;0\leq t\leq t_{R}$$ (8)

$$h(t)=kt_{R}=h_{max}\;t\geq t_{R}$$ (9)

Since the load, *F(t)*, *e*xponentially decays during the process, the solution is expressed as the step-loading relaxation solution adjusted by an RCF due to non-instantaneous ramp loading. Only the first two terms are considered for simplicity in calculations, as shown in Eqs 10 and 11 [23, 42]:

$$F(t)=A_{0}+A_{1}exp(-t/\tau_{1})$$ (10)

$$G(t)=B_{0}+B_{1}exp(-t/\tau_{1})$$ (11)

Where τ_1_ rpresents the time constant for the first exponential decay. *A*_*0*_ and *A*_*1*_, and *B*_*0*_ and *B*_*1*_ represent the fitting constants and the relaxation coefficients, respectively. Only the first term of both the fitting constants and relaxation coefficients (*i*.*e*. *A*_*0*_ and *B*_*0*_) are computed to compare the material relaxation in terms of applied force with the Wiechert theoretical model [43]. Once all the fitting parameters, *A*_*0*_ and *A*_*1*_, have been determined, they are converted to relaxation parameters, *B*_*0*_ and *B*_*1*_, using Eqs 12, 13 and 14, where t_r_ is time that it takes for the force to reach its maximum value:

B0=A0hmax3/2(8R3) (12)

B1=A1((RCF1)hmax3/28R3) (13)

RCF1=τ1tR[exp⁡(tR/τ1)−1] (14)

Instantaneous modulus, *E(0)*, can be computed from the fitted relaxation coefficients using Eq 15:

$$E(0)=E_{0}=1.5\;G(0)=1.5(B_{0}+B_{1})$$ (15)

The long term shear modulus, η, for each type of material can be calculated with Eq 16 [21]:

η=E·τ (16)

### Target preparation

The target preparation was divided into two parts: an investigation of 1) TMPs and 2) *ex vivo* hepatic and bile duct tissues in pre-clinical animal models. For the first part of this study, certain physical geometries and sizes of phantoms were used to satisfy homogeneity and isotropy assumptions for viscoelastic calculations. Additionally, flat, ideal-sized samples of animal tissues were chosen to avoid slippage of the indenter and to reduce any generated noise from the measurements for the second part of the study.

Three TMP types, agar, polyvinyl alcohol (PVA), and gelatin, were fabricated and investigated. These materials were chosen to mimic relevant human anatomical structures (*i*.*e*. prostate, oral cavity, liver, and breast) in terms of acoustic and mechanical properties [10–12, 18–21]. These water-based gels particularly satisfy the acoustic properties of human tissues, including the speed of sound (about 1540 m/s), attenuation (~0.5 dB^-1^ cm^-1^ MHz^-1^), and backscatter coefficient (between 10^−5^ and 10^−2^, between 2 and 7 MHz) [4].

These phantoms were also selected due to their distinguished elasticity ranges: PVA falls in the lower end of the elastic moduli spectrum at 1–40 kPa; gelatin is slightly higher at 10–100 kPa, and agar occupies the highest range at 100–200 kPa. In particular, 15% gelatin, 3% agar, and 17% PVA were fabricated due to their respective similarities to the acoustic velocity, acoustic impedance, and acoustic attenuation of ideal, healthy human tissue [4, 44–49].

For instance, the acoustic velocity in the PVA phantoms were shown to vary from 1520–1540 m/s, which is within the typical range for human soft tissue. Furthermore, PVA phantoms ranging between 14% to 20% are characterized by acoustic impedances similar to those of human breast and skin tissue. Agar TMPs, particularly within the range of 2% to 4%, match the acoustic properties of human prostate tissues. Lastly, the selected gelatin phantom concentrations mimic the acoustic attenuation of human tissues, specifically breast, liver, head and neck, and prostate [45–47, 50].

Agar (Agar, Sigma-Aldrich, St. Louis, MO) blocks of varying deionized water/agar concentrations were fabricated in a ~20 x 20 x 18 mm^3^ plastic mold. Three separate rectangular blocks of agar containing 2, 2.5, and 3%wt of agar were mixed and heated above their gel point (~90 ^o^C) to maximize cross-linking between the polymers. Increasing the amount of powdered extract in the mixtures was predicted to result in a corresponding increase in the elastic modulus of the phantom.

Gelatin (Porcine Gelatin, Sigma-Aldrich, St. Louis, MO) blocks of 10, 15, and 20%wt were used as a second type of TMP. Similar procedures as agar phantoms were used in the preparations of gelatin phantoms; however, the final mixture was placed in a centrifuge at a rotation speed of two rcf (relative centrifugal force) for a period of ~25 seconds to remove air bubbles from the solution.

Finally, a third type of TMP, PVA (99% hydrolyzed, Sigma-Aldrich, St. Louis, MO), was fabricated using the same procedure as stated above. Three cubic blocks (same dimensions as agar and gelatin phantoms) containing 14, 17, and 20%wt of PVA were synthesized. Unlike agar and gelatin phantoms, the final PVA mixtures were left at room temperature to cool down for ~two hours and then placed in a freezer at -20 ^0^C for a period of 24 hours. The phantoms were then taken out of the freezer and allowed to thaw at room temperature for ~two hours, completing one freezing-thawing cycle. All phantoms, agar, gelatin, and PVA, were made at least 90 minutes prior to measurements to avoid any confounding errors (*i*.*e*. dryness and degradation) in the collected data.

Fresh *ex vivo* liver and bile ducts were harvested from porcine and male Dewey rats shortly after each animal was euthanized with Isofluorane (5–30%, vapor only, no direct contact for rat) and intravenous injection of pentobarbital per weight for porcine. Approval was granted by UCLA institutional animal care and use committee (IACUC), protocols #2009-094-23 for rat tissues and #2003–093 for porcine tissues. The samples were stored in saline solution to avoid tissue dryness and degradation and maintain ideal physiological conditions during transportation to the measurement laboratory. Prior to measurements, the tissues were taken out of the saline solution and cut into smaller pieces measuring ~15 x 15 x 10 mm^3^ for porcine liver and slightly thinner for the other tissue types. After sectioning the samples, they were placed in a petri dish on a balance for viscoelastic measurements. Specimens were periodically moisturized using Ringer’s lactate solution to coat the specimen with isotonic fluid during the entire experimental procedure.
